# Supplementary material for: Transcriptomic and Metabolomic Insights into Benzylisoquinoline Alkaloid Biosynthesis in Goldthread (Coptis trifolia)
Source: Int J Mol Sci. 2025 Oct 5;26(19):9704. doi: 10.3390/ijms26199704 (PMC12525506; doi:10.3390/ijms26199704)
Supplement: Supplementary file 1 [file ijms-26-09704-s001.zip › ijms-3874290- supplementary figures.pdf]

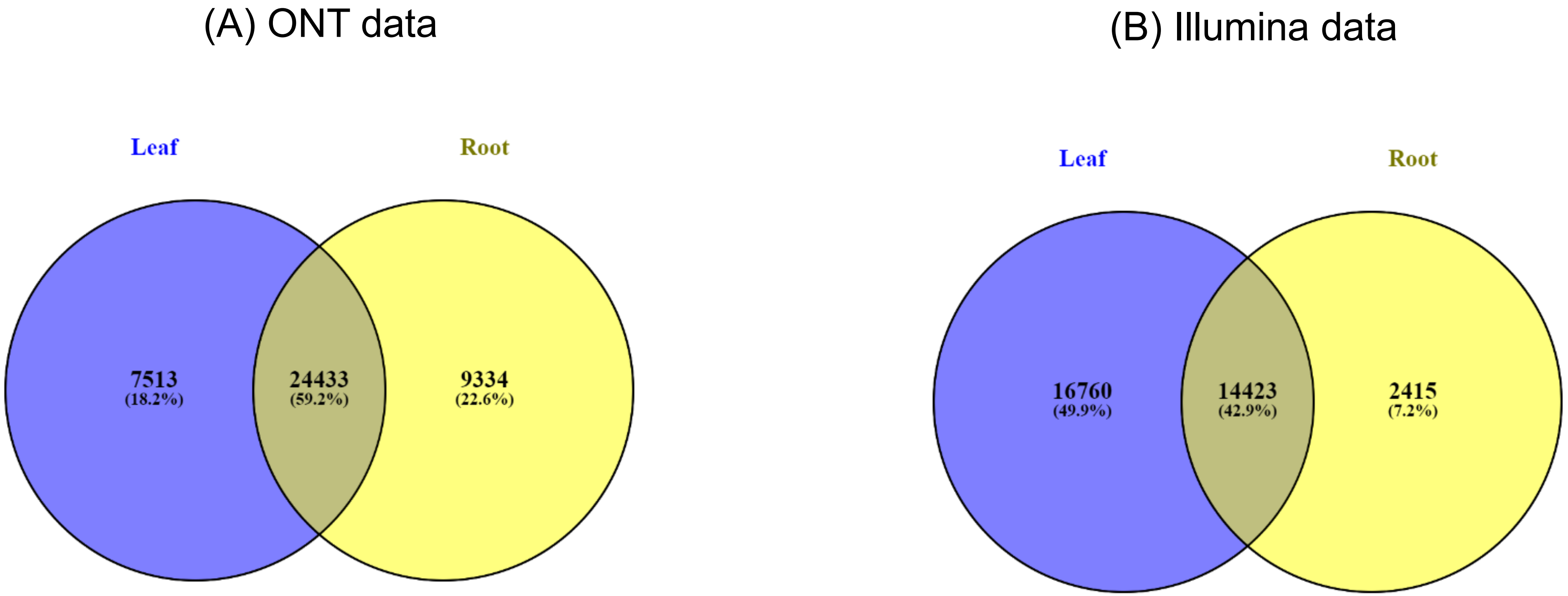

Figure S1: Comparison of assembled transcriptomes between leaf and root samples. (A) ONT long-reads data, (B) Illumina short-reads data

(A) PANTHER Overrepresentation Test of DE transcripts in leaf (left) and root (right)

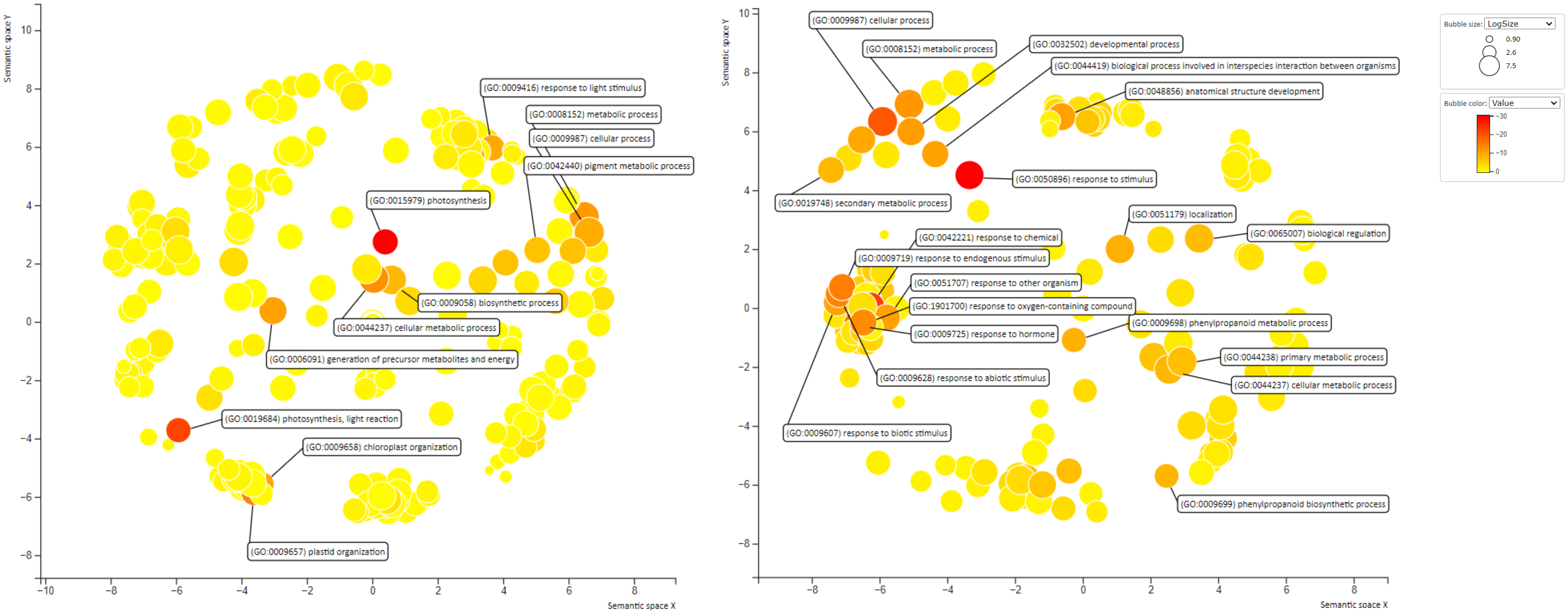

Figure S2: Functional enrichment test results. (A) PANTHER Overrepresentation Test of DE transcripts in leaf (left) and root (right), (B) Functional enrichment test in STRING of DE transcripts in leaf (left) and root (right), and (C) Functional enrichment test in STRING of DE transcription factors in leaf (left) and root (right).

(B) Functional enrichment test in STRING of DE transcripts in leaf (left) and root (right)

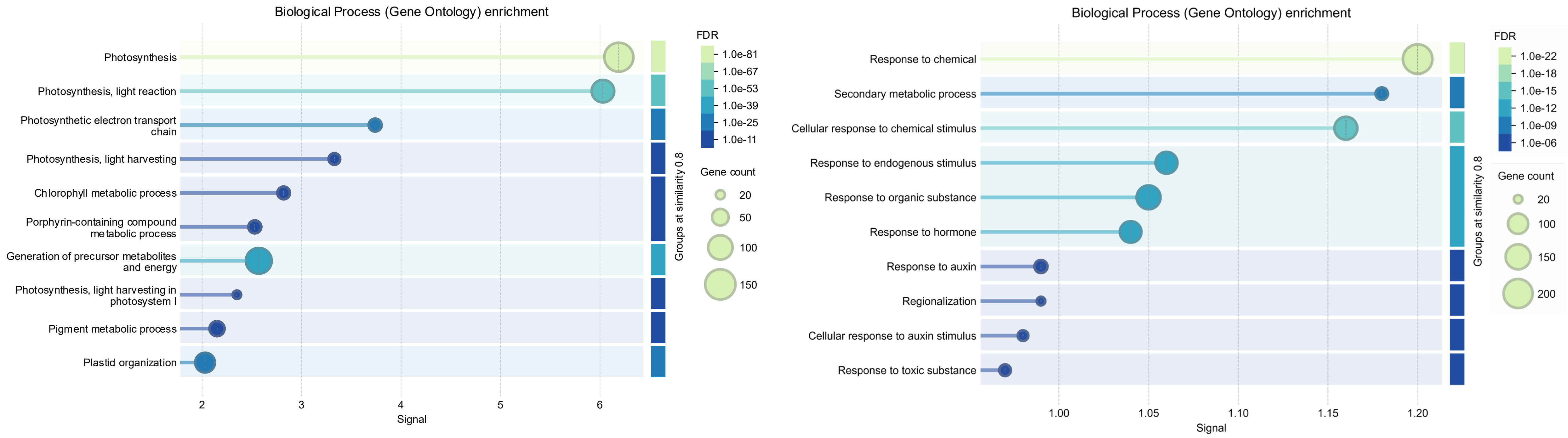

Figure S2. Continued.

(C) Functional enrichment test in STRING of DE transcription factors in leaf (left) and root (right)

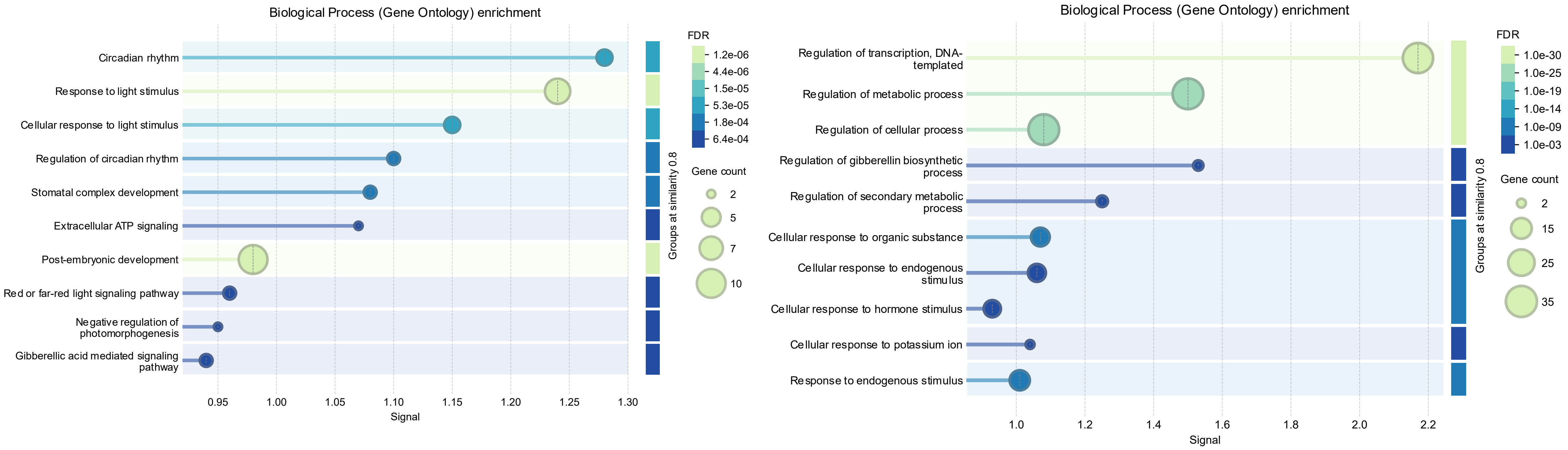

Figure S2. Continued.

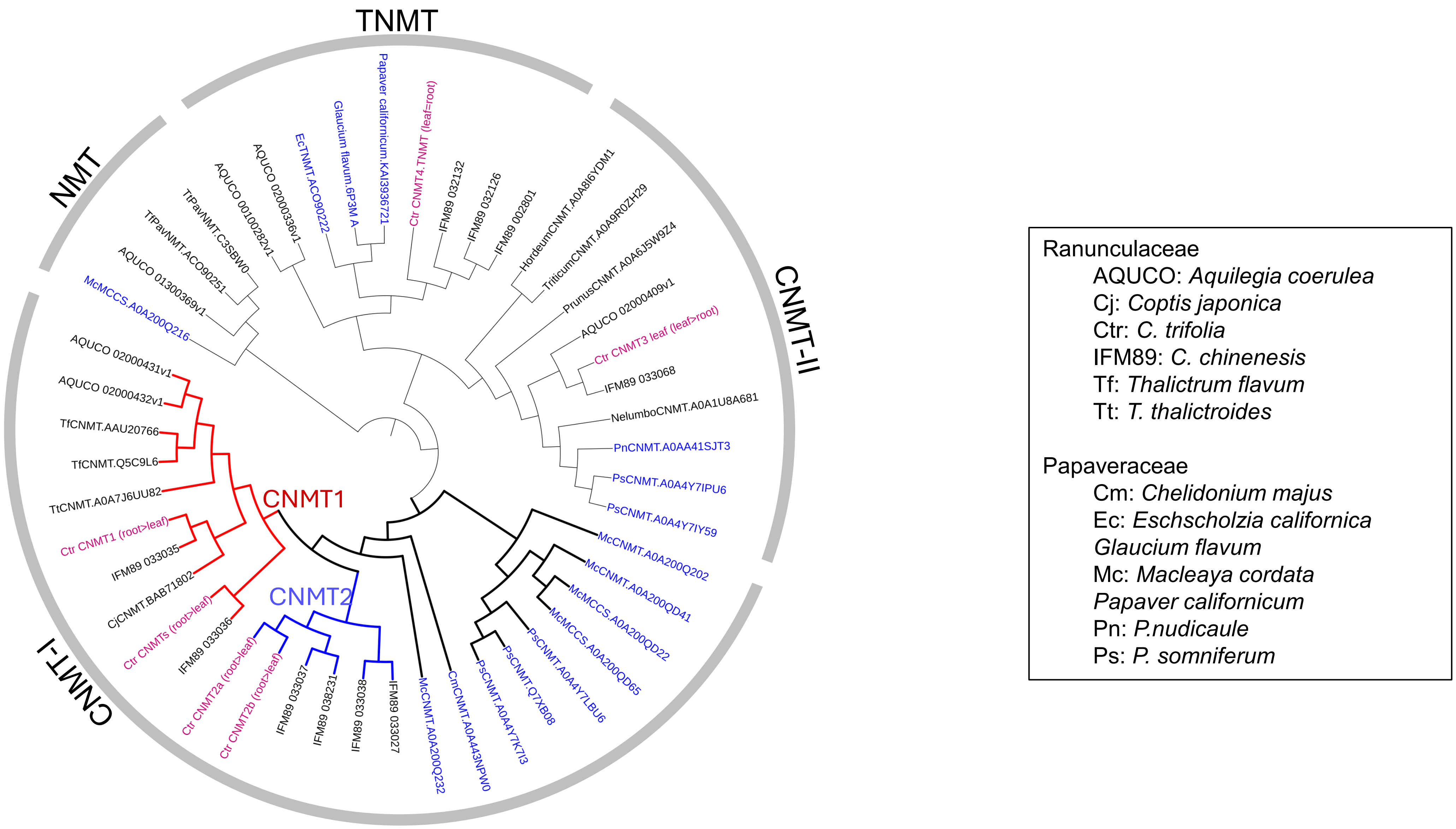

Figure S3: Neighbor-Joining tree of (S)-coclaurine N-methyltransferase (*CNMT*) gene homologs. The *N*-methyltransferase (*NMT*) clade was used as an outgroup. (*S*)-tetrahydroprotoberberine N-methyltransferase (TNMT) was included due to their close phylogenetic relationship to *CNMT*. Sequences from *C. trifolia* were shown in pink, with corresponding expression patterns. Sequences from Papaveraceae are labeled in blue.

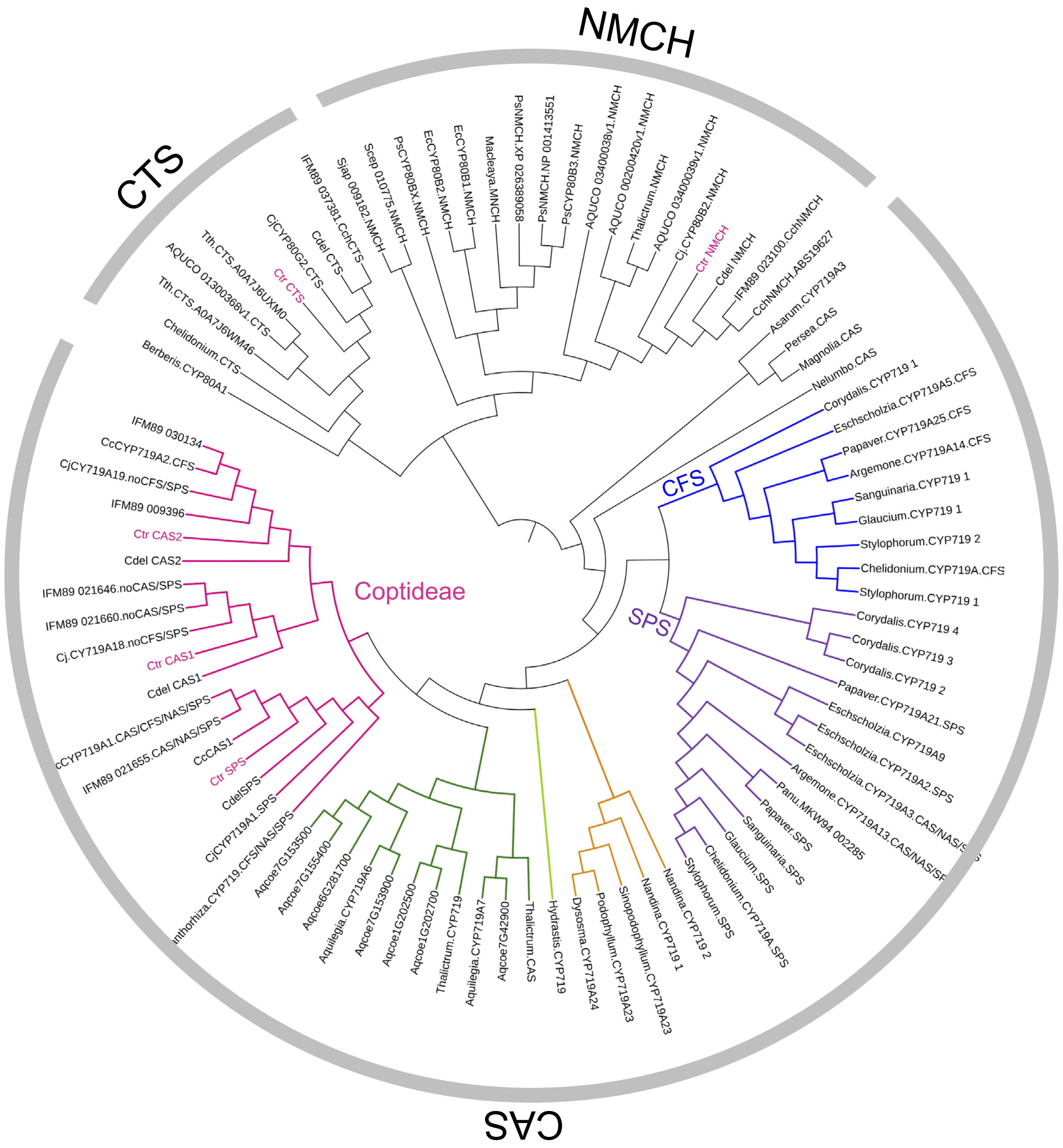

Figure S4: Neighbor-Joining tree of the cytochrome P450 members, (*S*)-corytuberine synthase (*CTS*, CYP80G), (*S*)-N-methylcoclaurine-3'-hydroxylase (*NMCH*, CYP80B), and (*S*)-canadine synthase (*CAS*, CYP719A). Sequences from *C. trifolia* were shown in pink. The information of sequences used in tree construction are provided in Table S3.

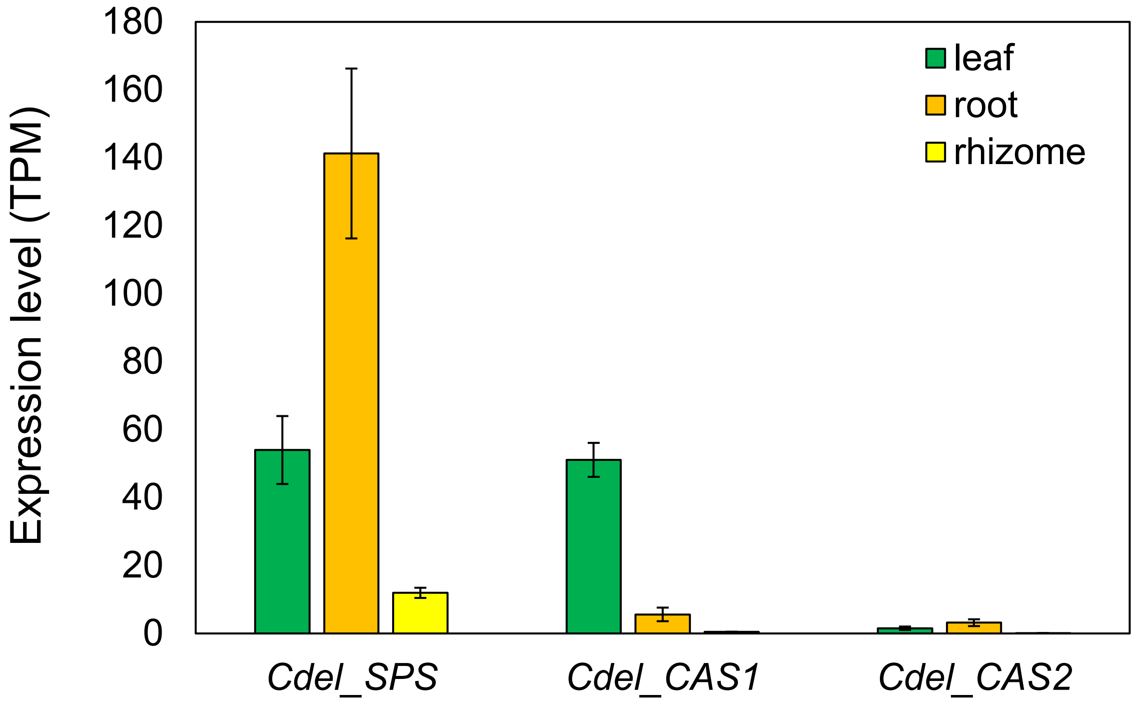

Figure S5: The expression levels of *CAS* homologs in *C. deltoidea* based on transcriptome data of Zhong et al. (2020)

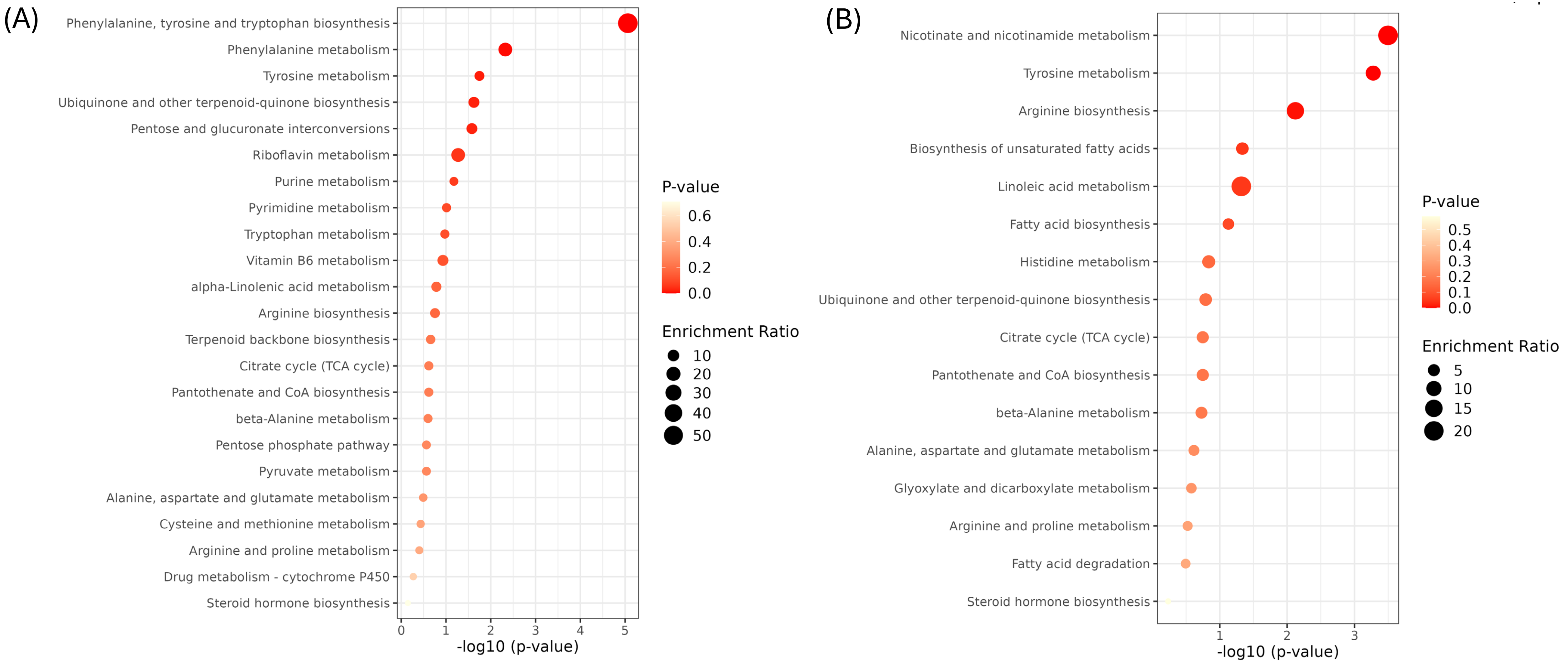

Figure S6: Pathway enrichment bubble plot of differentially accumulated metabolites in leaf (A) and root (B) tissues.

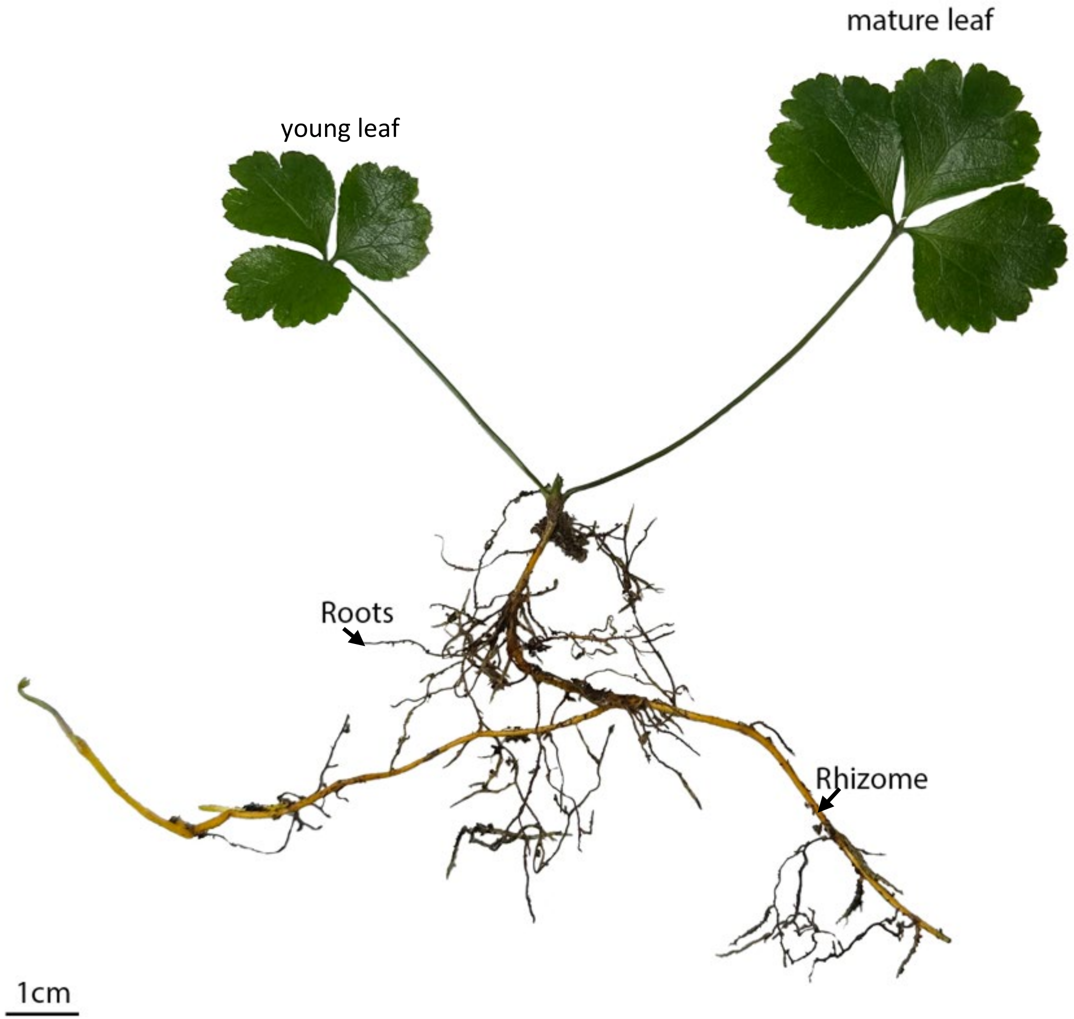

Figure S7: Image of *Coptis trifolia* used in this study. Mature leaf and roots with yellow rhizomes from two individuals were collected for each biological replicate.

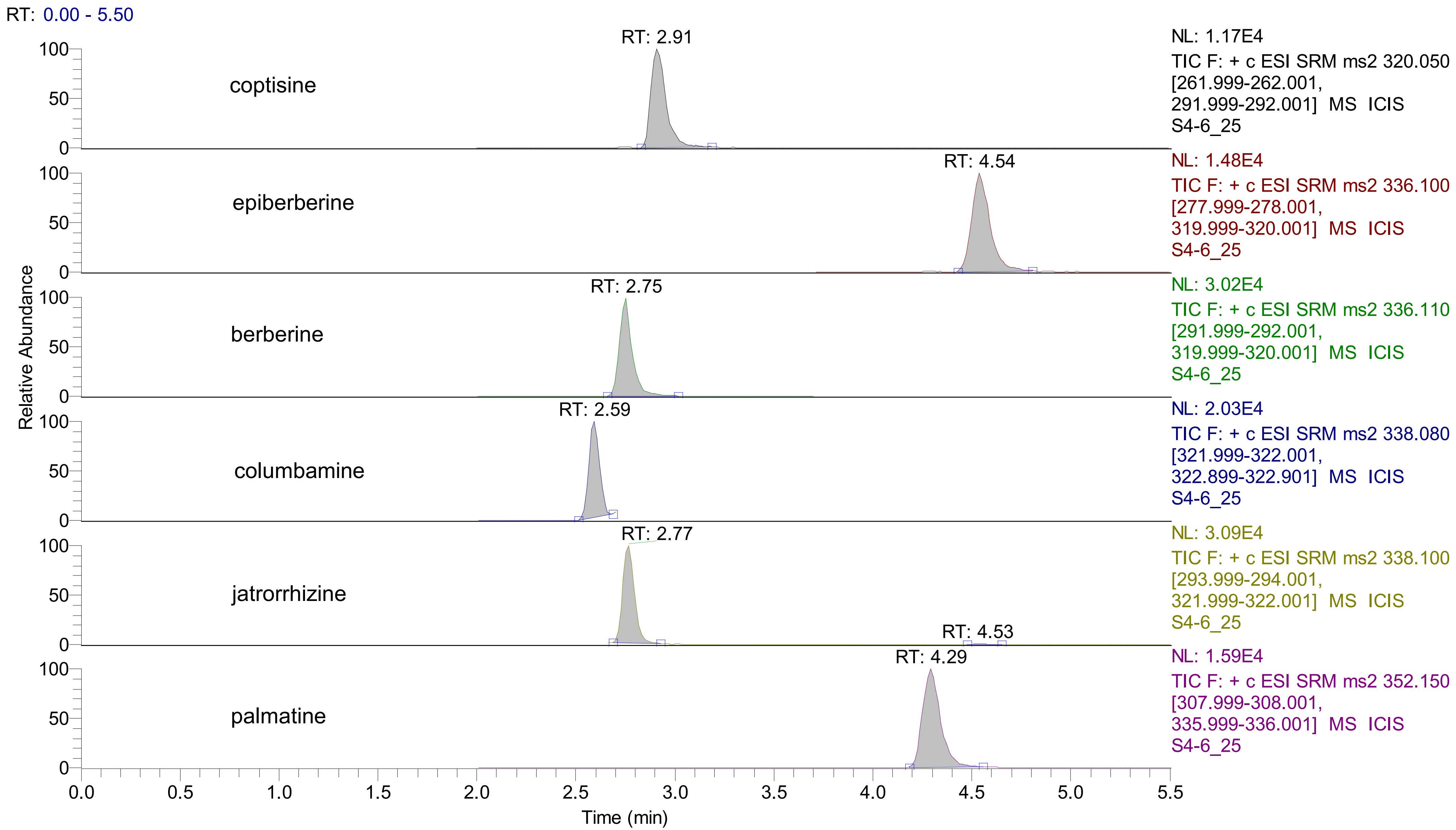

Figure S8: MRM chromatograms of coptisine, epiberberine, berberine, columbamine, jatrorrhizine, and palmatine.

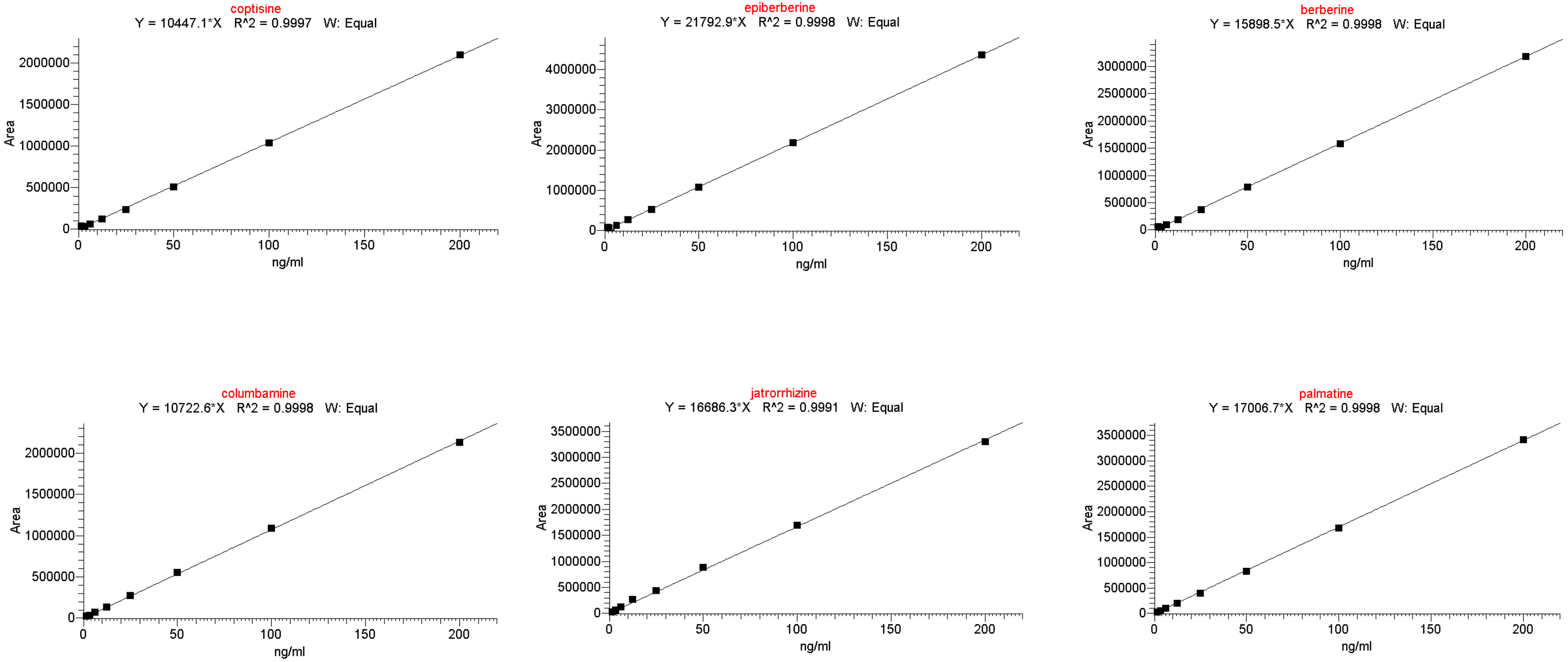

Figure S9: Calibration curves for standards of the six targeted metabolites.
